# RhVI1 is a membrane-anchored vacuolar invertase highly expressed in *Rosa hybrida* L. petals

**DOI:** 10.1093/jxb/erw148

**Published:** 2016-04-15

**Authors:** Domenica Farci, Gabriella Collu, Joanna Kirkpatrick, Francesca Esposito, Dario Piano

**Affiliations:** ^1^Laboratory of Plant Physiology and Photobiology, Department of Life and Environmental Sciences, University of Cagliari, Viale S. Ignazio da Laconi 13, 09123 Cagliari, Italy; ^2^European Molecular Biology Laboratory, Meyerhofstraße 1, 69117 Heidelberg, Germany; ^3^Laboratory of Molecular Virology, Department of Life and Environmental Sciences, University of Cagliari,Cittadella Universitaria di Monserrato, SS554, 09042 Monserrato, Cagliari, Italy

**Keywords:** Anthesis, bud, glycosylation, rose petals, senescence, sucrose, vacuolar invertases.

## Abstract

Invertases are a widespread group of enzymes that catalyse the conversion of sucrose into fructose and glucose. Plants invertases and their substrates are essential factors that play an active role in primary metabolism and in cellular differentiation and by these activities they sustain development and growth. Being naturally present in multiple isoforms, invertases are known to be highly differentiated and tissue specific in such a way that every isoform is characteristic of a specific part of the plant. In this work, we report the identification of the invertase RhVI1 that was found to be highly expressed in rose petals. A characterization of this protein revealed that RhVI1 is a glycosylated membrane-anchored protein associated with the cytosolic side of the vacuolar membrane which occurs *in vivo* in a monomeric form. Purification yields have shown that the levels of expression decreased during the passage of petals from buds to mature and pre-senescent flowers. Moreover, the activity assay indicates RhVI1 to be an acidic vacuolar invertase. The physiological implications of these findings are discussed, suggesting a possible role of this protein during anthesis.

## Abstract (precis)

RhVI1 is found to be a vacuolar membrane-anchored invertase that is highly expressed in buds. These findings suggest that RhVI1 has a role in blooming and corolla maturation by the regulation of sink-trafficking.

## Introduction

In the presence of sunlight and water, plants are able to reduce carbon dioxide into sugars, sustaining in this way their growth and development. These sugars, in particular sucrose, are partitioned between photosynthetically and non-photosynthetically active tissues (Tang *et al.*, 1999; Gonzalez et al., 2005; Sergeeva *et al.*, 2006; Wang *et al.*, 2010). Various pathways are actively involved in sucrose trafficking and partition, either between different subcellular compartments or between different plant districts (Sturm and Tang, 1999; Gibson, 2005; Wind *et al.*, 2010). In order to be used as a source of carbon and energy, sucrose must be hydrolysed to glucose and fructose. Invertases are enzymes that are able to cleave the oxygen bridge between two hexose units releasing them in their free form (Sturm, 1999). These enzymes are encoded by different genes and exist in several isoforms (Sturm, 1996; Tymowska-Lalanne and Kreis, 1998) which are known to be tissue-specific (Godt and Roitsch, 1997; Sturm *et al.*, 1999). They are characterized by different biochemical properties and subcellular localizations (Avigad, 1982), for example, cell wall and vacuolar invertases are characterized by an acidic pH optimum (pH 4.5–5.0) (Goetz and Roitsch, 1999) and are glycosylated in order to allow their transport to the final location/cellular compartment (Pereira *et al.*, 2014). However, the reason why specific invertase isoforms are coded for is not yet clear. Sugars in plants are important regulators of gene expression (Koch, 1996; Smeekens *et al.*, 2010; Wind *et al.*, 2010) and sucrose is essential for providing changes in osmotic pressure which thus provides a signal. Tissue-specific invertases may be indirectly involved in controlling cell differentiation and plant development in different organs (Ricardo and ap Rees, 1970; Wu *et al.*, 2005; Decourteix *et al.*, 2008; Bonhomme *et al.*, 2010) by affecting the signalling pathways and gene expression.

Analysing the amino acid sequence of the invertase family, two common features can be identified: the pentapeptide NDPNG, close to the N-terminus, and the highly conserved WECXDF sequence located closer to the C-terminus (Sturm and Chrispeels, 1990). Moreover, the vacuolar invertases also contain a short hydrophobic C-terminal sequence that could be involved in vacuolar targeting (Unger *et al.*, 1994). These enzymes are reported to be synthesized as pre-proenzymes, carrying an N-terminal sequence most likely composed of a signal peptide and a propeptide (Sturm and Chrispeels, 1990; Unger *et al.*, 1994; Sturm, 1999; Xiang and Van den Ende, 2013). The function of the N-terminal sequence is not clear, and it may play a role in protein folding, targeting and regulation (Hasilik and Tanner, 1987; Klionsky *et al.*, 1988; Rae *et al.*, 2011).

In most of the angiosperms, sucrose trafficking in the petals and, more generally, starch mobilization between sources and flowers, represent a metabolic signal which drives the flowers to the final blooming (van Doorn and van Meeteren, 2003). In *Rosa hybrida* L. petals, the mobilization of vacuolar starch (Ho and Nichols, 1977; Hammond, 1982) and sucrose trafficking (Yamada *et al.*, 2009) are shown to be essential for rose bud opening. Accordingly, it is not surprising that the enzymes related to carbohydrate mobilization and trafficking (amylases and invertases) are reported to be strongly involved in the complex processes of flowering in rose (Hammond, 1982; Sood *et al.*, 2006). Studies on *Rosa hybrida* L. showed that, from the action of these enzymes and their products, changes in osmotic pressure in different compartments, and particularly in the vacuole, lead to cell expansion which is an essential prerequisite for blooming (Yamada *et al.*, 2009).

In this study, we describe the purification of a membrane-anchored vacuolar invertase from petals of *Rosa hybrida* L. After the isolation of vacuolar membranes, a selective delivery of the protein *Rosa hybrida* Vacuolar Invertase isoform 1 (RhVI1) was achieved by solubilization using mild detergents. A subsequent step of size exclusion chromatography showed that the sample was highly pure and that the protein is a monomer. RhVI1 appears to have the typical invertase conserved sequences in the N- and the C-termini. From bioinformatic studies, it was shown that RhVI1 contains a signal peptide (for which it was not possible to determine a cleavage site) and a further transmembrane region. This observation is consistent with the solubilization experiments that suggested the membrane-anchored nature of the RhVI1 invertase. The physiological implications of these findings will be discussed.

## Materials and methods

### Plant material and growth conditions

*Rosa hybrida* L. was grown in the field or in glasshouses at a constant temperature of 25 °C under natural illumination and 60% relative air humidity.

### Membrane preparation and solubilization

Petal membranes were isolated at 4 °C in the dark by modifying a procedure for the isolation of thylakoid membranes from leaves (Berthold *et al.*, 1981; Haniewicz *et al.*, 2013). Briefly, 60g of petals from flowers at the same stage (buds or senescent flowers) were carefully removed from the flowers, washed twice in distilled water, dried in paper towels, weighed, mixed in a ratio 1:2 (g ml^–1^) with Grinding Buffer (GB: 50mM MES pH 6.5; 10mM MgCl_2_.6H_2_O; 10mM CaCl_2_.2H_2_O), and finally blended for 15s. The resulting suspension was filtered using two muslin layers with a layer of cotton between them. After filtration, the suspension was centrifuged at 5000 *g* for 10min at 4 °C and resuspended in a small volume of GB before being homogenized in ice. The resulting membranes were then solubilized for 20min with slow stirring at 4 °C in 20mM β-dodecylmaltoside (β-DDM) and subsequently centrifuged at 30 000 *g* for 10min at 4 °C. After centrifugation, the pellet was discarded and the supernatant, already significantly enriched in RhVI1, was used for further purification.

### Size exclusion chromatography

The solubilized membranes, already enriched in RhVI1, were diluted 50 times in order to bring the final DDM concentration to 0.4mM (0.02% m/v). This diluted pool was poured into an AMICON 9000 stirred cell, coupled to a 100kDa cut-off ultrafiltration membrane, and concentrated to a final volume of 10ml. Finally, the volume was further concentrated to 500 µl using a Vivaspin 20 ultraﬁltration with the same cut-off.

The protein sample was loaded on to a gel ﬁltration column (Superdex 200 10/30 GL, GE Healthcare) pre-equilibrated with gel filtration buffer [20mM MES–NaOH, pH 6.5; 5mM CaCl_2_; 5mM MgCl_2_; 10mM NaHCO_3_; 0.01% (w/v) β-DDM]. The sample, the main peak and the related fractions, was pooled and concentrated by ultraﬁltration (Vivaspin 20, 100kDa cut-off). The molecular weight of the RhVI1 was estimated by plotting the elution volume versus the logarithm of the molecular weight of the standard proteins (Gel Filtration Standard, Biorad) using a linear regression curve fit.

### Polyacrylamide Gel Electrophoresis

Denaturing SDS-PAGE was performed using 10% (w/v) separating polyacrylamide/urea gels with 4% (w/v) stacking gels, according to Schägger and Von Jagow (1987). The samples were denatured with Rotiload (Roth) at room temperature before loading and, after the electrophoretic separation, the gels were stained with Coomassie Brillant Blue G250 or silvered according to Switzer *et al.* (1979). For the glycoprotein staining assay, the Pro-Q Emerald 300 kit was used according to the manufacturer’s instructions (Molecular Probes). The molecular weight of the resolved RhVI1 was estimated by plotting the retardation factor values (*R* _f_, length of the band migration/length of the dye front) versus the logarithm of the molecular marker weights (Prestained Standard high range, Bio-Rad) using a polynomial regression curve fit.

### Mass spectrometry

The gel bands were excised from the SDS-PAGE and processed according to Farci *et al.* (2014) with a few modifications. Different SDS-PAGE bands were subject to independent digestions not only with trypsin, but also with LysC, GluC, chymotrypsin, and acidic hydrolysis. Data analysis was performed with the software MaxQuant (version 1.0.13.13) and the files obtained were used for searching in MASCOT (version 2.2.03, Matrix Science) against several plant species (ensembl Plants DB, NCBInr all species DB, including *Rosa hybrida* cvs) with a list of common contaminants appended.

### Activity assay

Functional tests were performed at 30 °C using the invertase assay kit MAK118 according to the manufacturer’s instructions (Sigma-Aldrich). A correction in the reaction buffer was introduced in order to perform the same measurement at different pH values. Functionality was assessed in the pH range 3–8. The level of activity was estimated by monitoring the levels of glucose produced. The glucose produced was expressed in µmol·min^−1^·mg^−^1.

### Bioinformatic analyses

The amino acid sequence of RhVI1 was analysed using several different software packages. The domains were identified using the InterPro EMBL-EBI Server (http://www.ebi.ac.uk/interpro/— Jones *et al.*, 2014; Mitchell *et al.*, 2015). The theoretical molecular mass was taken from the Uniprot Database (http://www.uniprot.org/— Magrane and UniProt Consortium, 2009). The theoretical isoelectric point was predicted using the Compute pI/MW ExPASy Server (http://web.expasy.org/compute_pi/l— Gasteiger *et al.*, 2005). The presence of transmembrane regions and orientation was predicted using the TMpred program (http://www.ch.embnet.org/software/TMPRED_form.html— Hofmann and Stoffel, 1993). The presence of the signal peptide and relative cleavage site was investigated using SignalP 4.1 Server (http://www.cbs.dtu.dk/services/SignalP/— Petersen *et al.*, 2011). Putative N- and O-glycosylation sites were predicted using the NetNGlyc 1.0 – CBS Server (http://www.cbs.dtu.dk/services/NetNGlyc/) and the NetOGlyc 4.0 – CBS Server (http://www.cbs.dtu.dk/services/NetOGlyc/— Steentoft *et al.*, 2013), respectively.

## Results

### Isolation of a dominant protein from the membrane fraction of petals

**Fig. 1.**
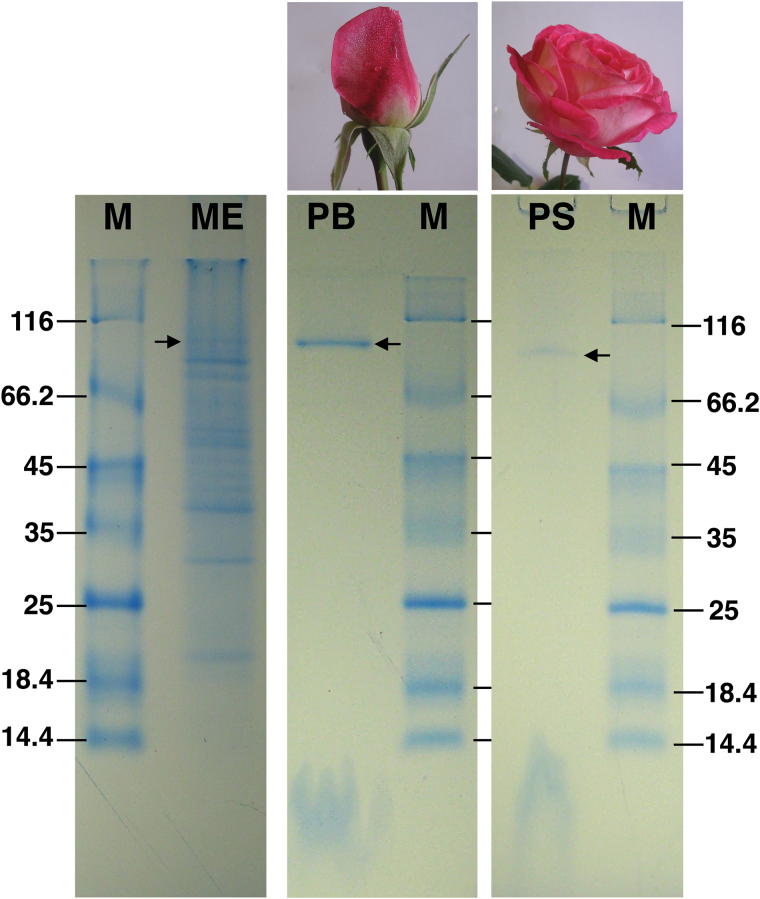
SDS-PAGE of isolated membranes (ME), solubilized membranes from buds’ petals (PB), and from senescent flowers’ petals (PS). After solubilization, in both cases a single protein is extracted showing that, in buds, the amount of protein isolated is significantly higher than in senescent flowers. Equal amounts of solubilized membranes were loaded for both PB and PS samples. The membrane stock was obtained starting from the same amount of flowers (see Materials and methods). The molecular marker is indicated by M. The arrows show the protein band in the three samples.

The whole membrane fraction from rose petals was isolated and characterized by SDS-PAGE (Fig. 1). These experiments not only led to a reproducible pattern of bands representative of the proteome associated with the petal’s membranes, but also, as expected, showed that, in these fractions, several proteins were more expressed when compared with the expression levels of the residual proteome components (Fig. 1). From several tests of membrane solubilization performed using different mild detergents of the alkyl-maltosides and glucosides series, it was found that β-dodecylmaltoside (β-DDM) was able to perform a selective solubilization of the membranes. Samples obtained by β-DDM solubilization were resolved by SDS-PAGE into a single band having an apparent molecular weight of ~90kDa. These results indicated that membrane solubilization leads to a sample specifically enriched in one of the proteins associated with the membrane fraction. Moreover, from the electrophoretic analysis, it also emerged that the expression level changed while the flower’s phenology evolved. This fact was evidenced by the presence of higher protein amounts in solubilized membranes from buds when compared with equivalent samples from mature or pre-senescent flowers (Fig. 1).

### The isolated protein occurs in a single oligomeric form

**Fig. 2.**
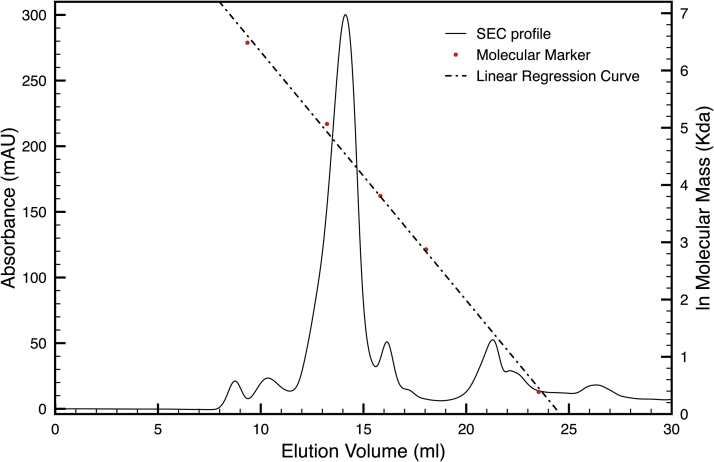
SEC profile of the extracted protein after buffer dilution and concentration. The sample separates into a dominant peak with minor impurities at lower masses. A polynomial regression curve is shown (dotted line) calculated on the basis of the retention volumes of the molecular standard (blue dots). The red dot on the polynomial regression curve shows the calculated mass of the isolated protein.

**Fig. 3.**
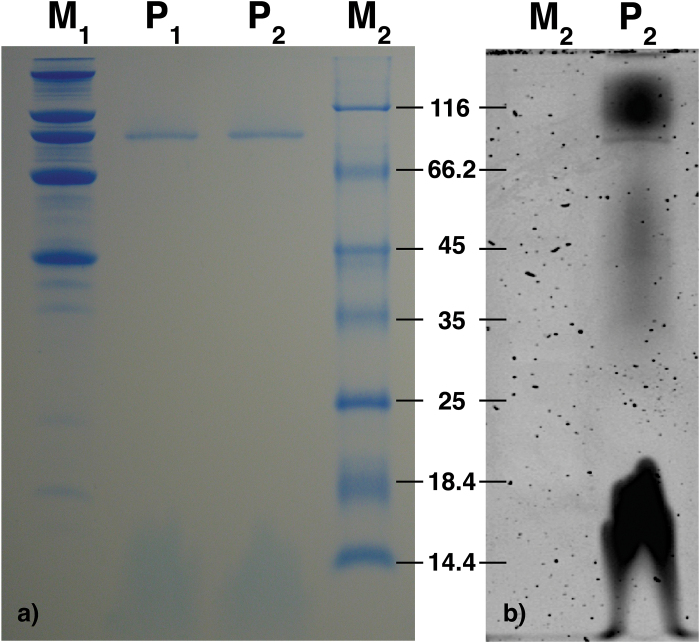
(a) SDS-PAGE of the two SEC fractions straddling the elution peaks (P_1_ and P_2_). The SEC separation leads to improved purity as can be seen by the presence of a single band with an apparent mass of ~90kDa. Two different molecular markers, indicated by M_1_ and M_2_, were used as a reference. (b) Glycoprotein staining assay on SDS-PAGE of the SEC fractions. The marker (M_2_), being composed of non- glycosylated proteins, is used as a negative control.

Protein isolation with high levels of purity had already been obtained after solubilization of the membrane fraction. In order to purify the protein further, identify its oligomeric profile, and define the precise mass, the protein samples were analysed by means of Size Exclusion Chromatography (SEC) (Fig. 2). From these experiments, the sample was resolved into a main peak which was re-analysed by SDS-PAGE (Fig. 3). The presence of a distinct dominant peak and of small amounts of other components proves the high level of protein purity found after the solubilization step. Moreover, the apparent mass associated with the main peak was calculated with respect to a linear regression curve based on a molecular standard. From this analysis, it was not only found that the unknown protein occurs in a single oligomeric state, but also that it has an experimental mass estimated by SEC and confirmed by SDS-PAGE of about ~93kDa and ~90kDa, respectively (Table 1).

**Table 1.** Masses of the cleaved signal peptide, of RhVI1, and of the sugar component calculated using different methods

Protein	Mass (kDa)	Determination method
Full length (theoretical mass)	65 375	Estimation from primary structure
Truncated and glycosylated (SEC experimental mass)	92 975	Linear regression
Truncated and glycosylated (SDS-PAGE experimental mass)	90 387	Polynomial regression
Truncated (MS mass)	52 912	MS
Signal peptide (MS mass)	12 463	MS
Sugars (calculated mass)	40 063	SEC/MS subtraction
	37 475	SDS-PAGE/MS subtraction

### The unknown protein is a vacuolar membrane-anchored invertase

**Fig. 4.**
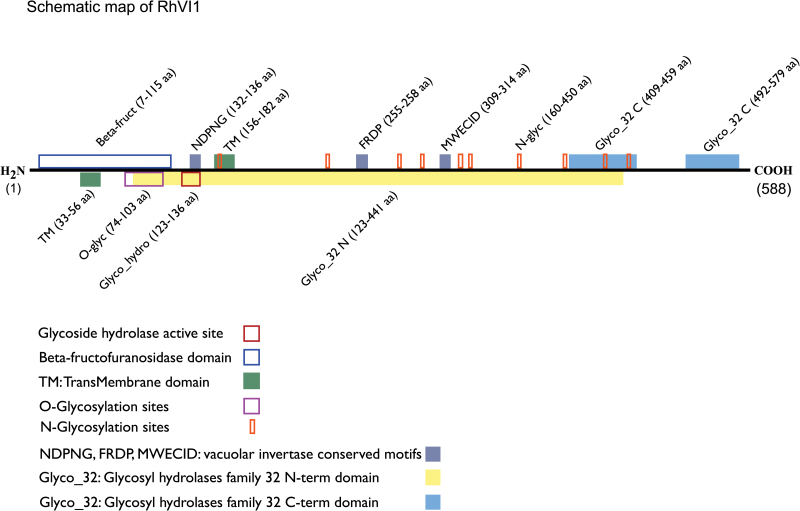
Schematic map of RhVI1 protein showing the main characteristic features in terms of sequence and specific regions. For details, see legend in the figure.

**Fig. 5.**
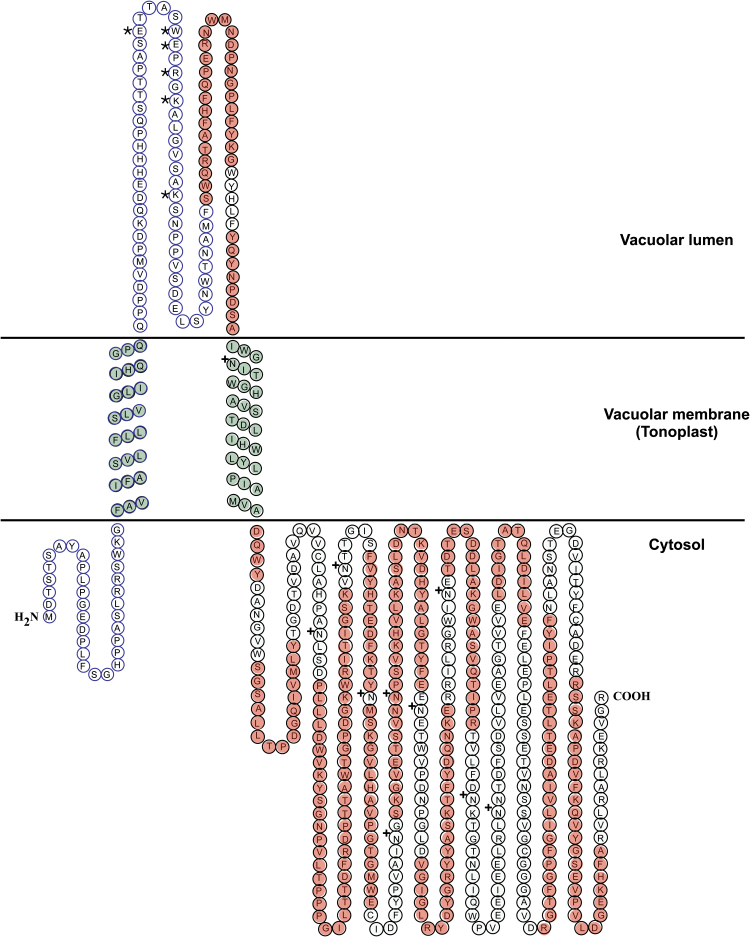
Scheme of RhVI1 with respect to its orientation in the vacuolar membrane. The peptide pattern, as obtained from the differential digestion and related MS analysis is shown in red. At the N-terminal side, the circles surrounded in blue represent the region not identified by MS while the part in light green indicates the two transmembrane regions one of which, the nearest to the N-terminal side, is the signal peptide; the sites of O-glycosylation and N-glycosylation are indicated by the symbols * and +, respectively.

Considering that the protein was isolated from the insoluble fraction of broken tissues and that the main source of membranes in the petals is the vacuole, the unknown protein was likely to be a vacuolar membrane protein. Further studies by Mass Spectrometry (MS) on the SDS-PAGE protein bands confirmed this hypothesis, leading to the identification of the unknown protein as RhVI1 (Table 2). We further confirmed the protein topology and localization by bioinformatic means. In particular, RhVI1 is found to have two N-terminal transmembrane regions, located between the first 200 residues for which the first transmembrane is predicted to be a signal peptide (Fig. 4; Fig. 5; see Supplementary Table S1 at *JXB* online). Moreover, several software packages for the compartmental localization of proteins confirmed, not only a possible association of RhVI1 to the vacuolar compartment of the cells, but also provided sufficient hints to assign the most likely orientation of the protein to the cytosolic side of the vacuolar membrane (Fig. 5). By analysing the complete protein sequence, we have also identified the highly conserved motifs NDPNG, FRDP, WECXDF, as well as the characteristic invertase domains (Supplementary Table S1). By the same means, in the N-terminal region we found a beta-fructofuranosidase domain (7–115 aa), which is involved in the hydrolysis of terminal non-reducing β-d-fructofuranoside residues, and a glycoside hydrolase active site (123–136 aa) containing an aspartic acid residue which is important for the catalytic mechanism. Finally, the major region of the protein is represented by the glycosyl hydrolase family 32 N-terminal (123–441 aa), that is the catalytic region and it is known to form a five bladed beta propeller structure to which are associated two glycosyl hydrolase family 32 C-terminal regions (409–459, 492–579 aa). Both of these regions are hypothesized to be involved in preserving the stability under high temperature and are known to form a beta sandwich module (Fig. 4; Supplementary Table S1).

**Table 2.** MS analysis and protein identification on the basis of differential digestion using four different types of proteases and acidic hydrolysis

	Digestion type				
	Trypsin	Chymotrypsin	GucC	LysC	Acid hydrolysis
Sequence coverage (%)	15	22	20	23	29
Highest score	715	856	695	973	1861
Queries matched	38	37	21	55	85
emPAI	0.63	0.86	0.41	0.88	0.34
Highest score identification	Vacuolar invertase isoform 1 (*Rosa hybrida* L.)	Vacuolar invertase isoform 1 (*Rosa hybrida* L.)	Vacuolar invertase isoform 1 (*Rosa hybrida* L.)	Vacuolar invertase isoform 1 (*Rosa hybrida* L.)	Vacuolar invertase isoform 1 (*Rosa hybrida* L.)

### RhVI1 is a glycosylated protein

The measured mass, identified either by SEC and/or by SDS-PAGE, is of about ~90kDa, while the theoretical mass for this protein is ~65kDa (Figs 3, 4). This significant difference between the calculated and the measured mass suggested possible post-translational modifications on the monomers rather than some higher oligomeric form, such as dimers. In order to identify any possible site of post-translational modification which could account for the observed mass discrepancy, a bioinformatic analysis was performed, leading to the identification of the O- as well as the N-glycosylation sites (Supplementary Table S1). The MS analysis, performed using five different digestion strategies, led to a sequence coverage of ~51%, thus confirming the first 420 C-terminal residues (red residues in Fig. 5). However, if, from the C-terminal side, the coverage is homogeneously distributed, the first 115 N-terminal residues are fully uncovered strongly suggesting the cleavage of the predicted signal peptide region (residues surrounded in blue, Fig. 5) and allowing the hypothesis that the protein can be subject not only to O-, but also to N-glycosylation (respectively * and + symbols in Fig. 5). From the combination of these results, it emerged that the loss of 115 residues, equivalent to ~12.5kDa, leads to a decrease in the theoretical protein mass from ~65kDa to ~52.5kDa. Considering that the mass calculated by SEC and SDS-PAGE is of 90 and 93kDa, respectively, it can be concluded that the contribution of the sugars, with respect to the total protein weight, would be 37.5–40.5kDa (Table 1), equivalent to ~41% of the protein mass.

In order to prove or disprove this hypothesis, the RhVI1 resolved by SDS-PAGE (Fig. 3) was processed using a sensitive glycoprotein staining assay (Pro-Q Emerald 300, Molecular Probes) and compared with the same gel stained by Coomassie Brilliant Blue G250 and silvering (see Supplementary Fig. S1 at *JXB* online). These experiments confirmed that RhVI1 is a glycosylated invertase.

### RhVI1 is an acidic invertase

**Fig. 6.**
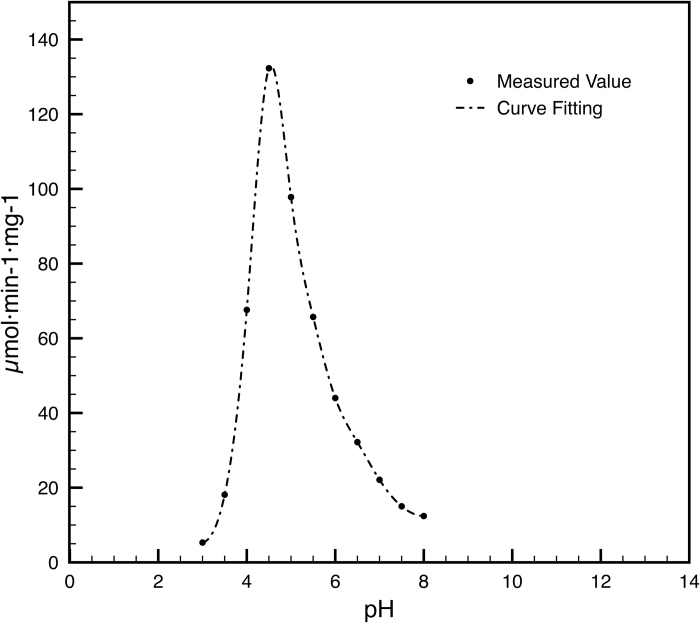
RhVI1 activity assay with respect to different pH tested in a range between 3 and 8 pH units.

Functionality assays, performed on the SEC pool, showed higher activity in acidic conditions with an optimum at pH 4.5 (Fig. 6). This is consistent with the observation that this class of enzymes possesses an optimal pH in the same order as its pI (Cherry *et al*., 1999) that, in the case of RhVI1, was calculated to be 4.78. This property, as expected for a vacuolar invertase, confirmed that RhVI1 is acidic with activity rates consistent with other similar acidic invertases.

## Discussion

Plant invertases are mostly known as soluble proteins involved in several aspects of energetic metabolism (Godt and Roitsch, 1997), with particular involvement in photosynthetic efficiency and regulation (Büssis et al., 1997). In this study, we report the isolation and characterization of the membrane-anchored vacuolar invertase RhVI1 from petals of *Rosa hybrida* L. In previous work, it was shown that the expression of *RhVI1* changes in buds under different illumination regimes and that *RhVI1* codes for an enzyme with vacuolar localization on the basis of its sequence (Girault *et al.*, 2010; Rabot *et al.*, 2014). This suggested a physiological significance of RhVI1 in processes such as anthesis where a high responsiveness to light and sugars is required (Bartlett and Remphrey, 1998; Niinemets and Lukjanova, 2003; Kawamura and Takeda, 2004; Girault *et al.*, 2008; Henry *et al.*, 2011). Gibberellins are a class of phytohormones that play a primary role in anthesis induction and sustainability (Fos *et al.*, 2000). Consistent with this, *RhVI1* expression was found to be strictly dependent on gibberellins which were shown to enhance the expression of this invertase gene on its promotor (Choubane *et al.*, 2012; Rabot *et al.* 2014). In this context, *RhVI1*, modulated by gibberellins, behaves as an essential effector that acts as a bridge between the primary metabolism and the environmental stimuli (Choubane *et al.* 2012; Rabot *et al.* 2014). Most likely, as observed for other invertases (Glasziou and Gayler, 1972; Evert, 1982), RhVI1 allows fast growth of the tissues affecting hexose transport which, in this case, results in the promotion of anthesis (Koonjul *et al.* 2005; Morley-Smith *et al.* 2008). Consistently, in this work we not only isolate and characterize RhVI1, but we also observe a specific profile of RhVI1 levels with respect to the phenology of *Rosa hybrida* L. flowers. In particular, we observe a quantitative decrease of this vacuolar enzyme as buds change to senescent flowers. This experimental evidence confirms a role of invertases in promoting growth in sink organs by increasing the hexose levels through the degradation of their substrate—sucrose. Similar observations have already been reported for flower-specific invertases in *Dacus carota* L. (Lorenz *et al.* 1995), *Lycopersicum esculentum* L. (Godt and Roitsch, 1997), and *Arabidopsis thaliana* L. (Tymowska-Lalanne and Kreis, 1998). However, in all these cases, only soluble invertases were found.

So far no evidence has been reported for a membrane-anchored vacuolar invertase and its presence in petals still remains unclear. However, several aspects could contribute to provide some clues. RhVI1 is identified as a membrane protein with two transmembrane regions. The first 115 residues at the N-terminal side contain one of the transmembrane regions which is predicted to be the signal peptide and for which it was not possible to identify a possible cleavage site. However, the differential digestion patterns and the associated MS analysis provided a protein sequence where the predicted signal peptide region is missing, suggesting a loss of this part during processing of the protein (Figs 4, 5). This loss appears to be a typical feature of vacuolar invertases which, in their mature forms, are frequently truncated and glycosylated (Sturm, 1999). Glycosylation is a feature that characterizes RhVI1 and, in this work, was attributed by indirect evidence such as the mass estimation (Figs 2, 3) and prediction from the bioinformatic analysis (Figs 4, 5), and by direct evidence of the glycoprotein staining assay (Fig. 3).

RhVI1 was selectively extracted from the membrane fractions as it showed a particular affinity for mild detergents. This detergent-dependent selectivity is not unusual for the isolation of membrane proteins (Haniewicz *et al.* 2015). There was no trace of RhVI1 in the hydrophilic fraction of the crude extract thus confirming the membrane protein properties of this enzyme. The assumption that RhVI1 has two transmembrane regions, one of which is cleaved and is a signal peptide, indicates the unusual association of this invertase with the vacuolar membrane, and also the fact that the whole protein, through the C-terminal side, would be exposed to the cytosolic face (Fig. 5). These observations contrast with the RhVI1 optimal pH (4.5) and pI (4.78), which are both typical for a vacuolar invertase. In a recent report, Rabot *et al.* (2012) observed that, in bursting buds, *RhVI1* expression was up-regulated when compared with its isoform *RhVI2*, which was down-regulated. This fact would suggest a correlated function between these enzymes. In particular, it indicates the presence of factors that affect the expression and, most likely, the activity of these two isoforms in an opposing way. Following the same logic, we also observed that RhVI2 is a membrane protein (see the Materials and methods) which has a transmembrane region, predicted to be a signal peptide in the N-terminal side, and a second transmembrane region at the C-terminal side. This means that, after the loss of the signal peptide, the protein will stay anchored to the vacuolar membrane on its vacuolar side. Again, it can be hypothesized that these two isoforms have opposite features, since the vacuolar membrane invertase RhVI1 faces the cytosolic side and has a pI of 4.78, while the vacuolar membrane invertase RhVI2 faces the vacuolar side and has a pI of 6.29. Invertases are known to be strictly dependent on pH and, as confirmed in this work for RhVI1, their optimal activity is near to their pI (Cherry *et al*., 1999). What has been observed is that the optimal pH activities for both enzymes differ significantly with respect to the typical pH range of the compartment in which they are located. This apparent contradiction would favour the hypothesis that RhVI1 and RhVI2 activities are subject to factors which induce opposite effects on these two isozymes, reinforcing the idea that both isoforms may be part of a system able to regulate the sucrose levels and trafficking between the vacuole and the cytosol. Sucrose trafficking across the vacuolar membrane is strictly dependent on the proton-sucrose antiporter (Wormit *et al.* 2006). This system requires fine regulation in order to avoid an unbalanced sucrose concentration between the two compartments (Etxeberria *et al.*, 2012). Having opposite functional features and localizations, the two isoforms could provide a regulation system of the sucrose influx/efflux across the vacuolar membrane by sensing the associated local pH changes leading to the activation/inactivation of these two enzymes. Sink organs, such as buds, are characterized by a preferential sucrose supply (Lemoine *et al.*, 2013). Part of the sucrose will be used for metabolism, while the rest will be subject to short-term storage in the vacuole (Endler *et al.*, 2006). These two possible destinations leads to distribution between cell compartments which will keep changing through a steady-state equilibrium depending on several exogenous and endogenous factors that finally affect the metabolic rates.

Levels of cytosolic sucrose above a given threshold promote vacuolar storage (Etxeberria and Gonzalez, 2001). This activates the proton-sucrose antiporter which, while pumping sucrose in, pumps out vacuolar protons to the cytosol (Wormit *et al.*, 2006). This mechanism brings about a decrease in the local pH of the cytosolic side and a pH increase in the vacuolar side of the membrane. This movement of protons and sucrose across the vacuolar membrane would activate both RhVI1, decreasing vacuolar intake, and RhVI2, promoting vacuolar hexose production which would ultimately lead to the cessation of sucrose storage. Conversely, under low sucrose availability, the local pH of both sides is far from the optimal activity of the two invertases, a fact that will allow the promotion of sucrose storage.

An important observation that arises from our analysis is the absence of RhVI2 in our preparations. RhVI2 was not even observed in our MS analysis suggesting that, in general, a low number of copies of this enzyme are present, confirming previous results (Rabot *et al.*, 2012). However, it must be mentioned that the vacuolar orientation of RhVI2 does not require its presence in large copy numbers when compared with RhVI1 which, by contrast, is affected by rapid pH stabilization due to the more complex cytosolic composition and extension. Couples of isoforms homologous to this system are observed in other species of the Rosaceae and the presence of two isoforms homologous to *RhVI1* and *2* has also been found in monocotyledons, such as *Zea mays* L. (Supplementary Table S2 ). This fact suggests, not only that this association is common in plants, but also that it may be part of the basic plant cell regulation mechanisms.

Sucrose represents a main source of carbon and energy which are both important factors in sink organs, so this carbohydrate is one of the most concentrated metabolites in vacuoles, especially in petals (Yamada *et al.*, 2007). The solute content in the vacuole is essential for regulating the turgor state of the cells so sucrose also plays an essential role in determining the turgor state of the tissues (Perry *et al.*, 1987). Fine regulation of the turgor is needed in plant growth (Geitmann and Ortega, 2009; Hamant and Traas, 2010; Robinson *et al.*, 2013) and at specific periods of the biological cycle such as anthesis (Yamada *et al.*, 2007; Beauzamy *et al.*, 2014). It is known that the turgor pressure is mostly against the membranes and an increase in hydraulic pressure is often associated with an increased cytoplasmic concentration (Van der Berg et al., 2000, Ellis, 2001; Briegel *et al.*, 2014). Solutes can be moved across different compartments only if the transporters and their regulators will allow the transport (Etxeberria *et al.*, 2012). Since the membranes can be affected by pressure (Beauzamy *et al.*, 2014), the presence of a membrane-anchored invertase system could be useful in order to sense the sugar concentration. This would immediately activate the store of sugars inside the vacuole, in the case of increasing cytoplasmic concentration or, conversely, move them to the cellular cytoplasm in the case of a decrease.

Anthesis represents the stage in which flowers reach its structural and functional maturity (Kirchoff and Claβen-Bockhoff, 2013). During this period a complex sequence of chemical–physical processes take place that were shown to be partially dependent on the invertases activity (Koonjul *et al.*, 2005). In particular, flower maturation and blooming were found to be related to the vacuolar invertases of petal cells (Moghaddam and van den Ende, 2013). In *Rosa hybrida* L., during petal growth, vacuolar invertases drive the production of monosaccharides from sucrose, leading to an increase in osmotic pressure. This mechanism is responsible firstly for petal cells expansion leading to petal growth (Yamada *et al.*, 2009) and then for the corolla maturation and flower opening (van Doorn and van Meeteren, 2003). These processes are mediated by two isoforms of vacuolar invertase, RhVI1 and RhVI2 (Rabot *et al.*, 2012). In the present work, we describe RhVI1 as a vacuolar membrane-associated invertase and RhVI2 as a invertase putatively associated to the vacuolar membrane. Considering their peculiar topology and reciprocal orientation, the co-ordination between *RhVI1*and *RhVI2* may play a key role in sustaining corolla maturation, not only bringing nutrients to the flower, but also supporting the blooming mechanisms by providing attraction as well as access to pollinators.

## Supplementary data

Supplementary data can be found at *JXB* online.

Figure S1, SDS-PAGE on the SEC fractions stained by Coomassie Brilliant Blue G250 (a), silvering (b), and the glycoprotein staining assay (c).

Table S1, Main regions and domains of the RhVI1 and their related functions.

Table S2. Comparison between the homologues of *RHVI1* and *2* with two other dicotyledons species from the Rosaceae and also with the monocotyledon *Zea mays* L. (Graminaceae).

Supplementary Data
