# Supplementary material for: RhVI1 is a membrane-anchored vacuolar invertase highly expressed in Rosa hybrida L. petals
Source: J Exp Bot. 2016 Apr 15;67(11):3303–12. doi: 10.1093/jxb/erw148 (PMC4892724; doi:10.1093/jxb/erw148)
Supplement: Supplementary Data [file supp_67_11_3303__index.html]

RhVI1 is a membrane-anchored vacuolar invertase highly expressed in Rosa hybrida L. petals — RhVI1 is a membrane-anchored vacuolar invertase highly expressed in Rosa hybrida L. petals — Supplementary Data 

# RhVI1 is a membrane-anchored vacuolar invertase highly expressed in *Rosa hybrida* L. petals

## Supplementary Data

Data files

- supplementary\_figure\_S1\_tables\_S1\_S2.pdf - Supplementary Data
