# Supplementary material for: RhVI1 is a membrane-anchored vacuolar invertase highly expressed in Rosa hybrida L. petals
Source: J Exp Bot. 2016 Apr 15;67(11):3303–12. doi: 10.1093/jxb/erw148 (PMC4892724; doi:10.1093/jxb/erw148)
Supplement: Supplementary Data [file supp_erw148_supplementary_figure_S1_tables_S1_S2.pdf]

Supplemental Files

Supplementary figure 1: SDS-PAGE on the SEC fractions stained by Coomassie (a), silvering (b) and the glycoproteins staining assay (c). In the lanes 1, 2, 3 and 4 are loaded 0.4, 0.3, 0.2, 0.1 μg of RhVI1, respectively. The asterisks (*) show the migration level of the RhVI1 band. M is indicating the lane with the molecular marker.

Supplementary table 1:
Main regions and domains of the RhVI1 and their related functions.

Supplementary table 2:
Comparison between the homologues of *RHVI1* and *2* with two other dicotyledons species from the same family (*Rosaceae*) and also with the monocotyledon *Zea mais* L. (*Graminaceae*). The level of homology and the difference in pI values between the two isoforms are conserved suggesting a basic mechanism related to their function.

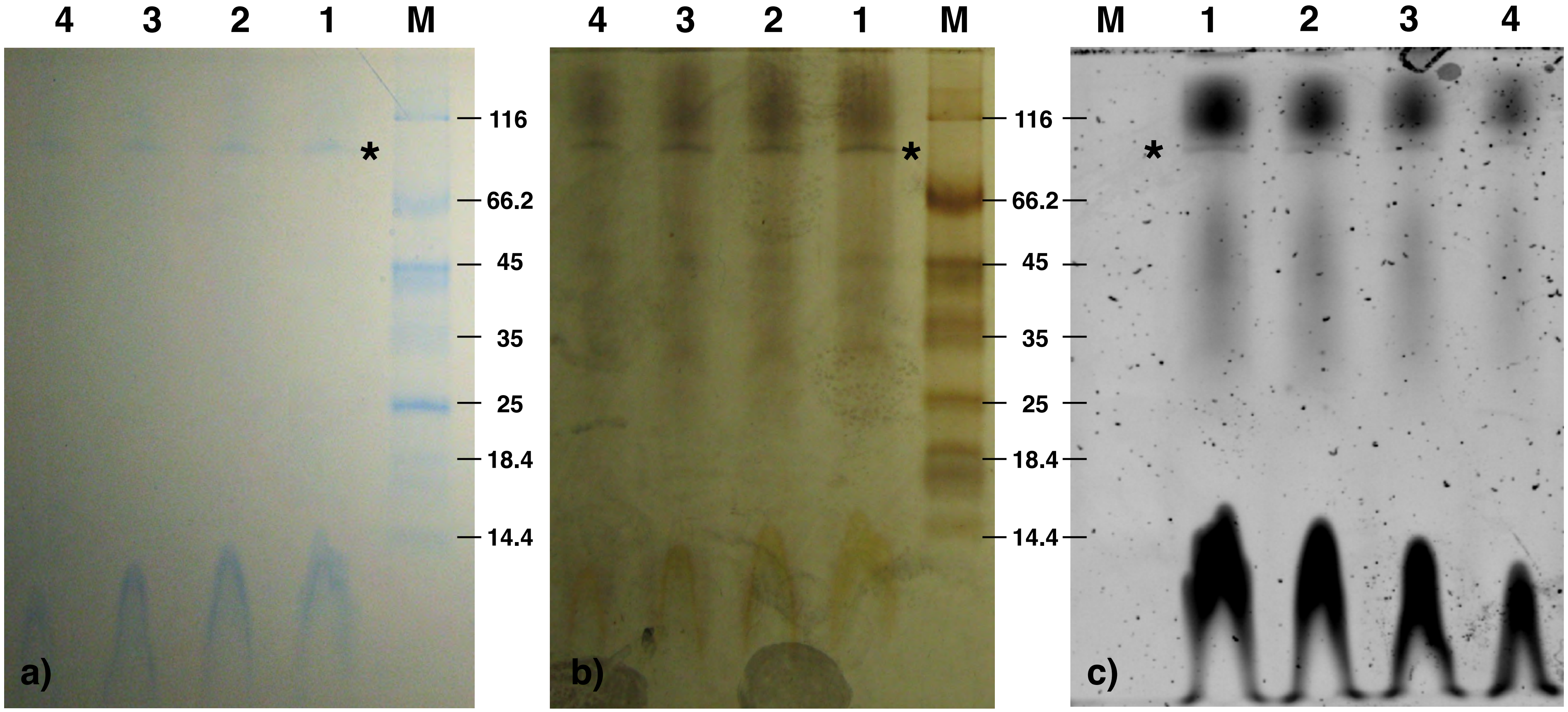
4 3 2 1 M
116
66.2
45
35
25
18.4
14.4
*
a)
4 3 2 1 M
116
66.2
45
35
25
18.4
14.4
*
b)
M 1 2 3 4
*
c)

Domain Name	Amino Acid Residues	Function	Software
Beta-fructofuranoside	7-115	hydrolyses the terminal non-reducing beta-D-fructofuranoside residues	InterPro EMBL-EBI
Transmembrane	33-56 , 156-182	anchors the protein to the vacuole membrane	InterPro EMBL-EBI
O-Glycosylation	74-103	involved in protein stability and folding	NetOGlyc 4.0 - CBS
N-Glycosylation	160-450	involved in protein stability and solubility	NetNGlyc 1.0 - CBS
Glycoside hydrolase active site	123-136	contains an aspartic acid residues important for the catalytic mechanism	InterPro EMBL-EBI
Glycosyl hydrolase family 32 N-terminal	123-441	catalytic region, forms a five bladed beta propeller structure	InterPro EMBL-EBI
Glycosyl hydrolase family 32 C-terminal	409-459 , 492-579	a role in preserving stability at high temperature is hypothesized, forms a beta sandwich module	InterPro EMBL-EBI

Specie	Gene name	Transmembran e domain	Theoretical pI	Homology	Site of vacuolar membrane localization
**Rosa hybrida L.**	RhIV1 (H2DF87)	TM 32-1	pI 4.78	-	Cytoplasmic
	RhIV2 (H2DF88)	TM 30-49 / 617-639	pI 6.29	-	Vacuolar
**Pyrus pyrifolia L.**	PpSAIV1 (A0A7Y9)	TM 32-51	pI 4.98	Homology with RhIV1 - 75%	Cytoplasmic
	PpSAIV2 (A0A7Z0)	TM 39-59 / 661-680	pI 5.87	Homology with RhIV2 - 75%	Vacuolar
***Prunus persica* L.**	PRUPE1 (M5XEX0)	TM 30-49	pI 4.75	Homology with RhIV1 - 74%	Cytoplasmic
	PRUPE2 (M5WQD6)	TM 666-685	pI 6.45	Homology with RhIV2 - 78%	Vacuolar
***Zea mays* L.**	IVR1 (P49175)	TM 44-66	pI 5.06	Homology with RhIV1 - 55%	Cytoplasmic
	IVR2 (Q7XTK7) --fragment-	n.d.	pI 4.88	Homology with RhIV2 - 65%	Vacuolar
